# ViralFlow v1.0—a computational workflow for streamlining viral genomic surveillance

**DOI:** 10.1093/nargab/lqae056

**Published:** 2024-05-25

**Authors:** Alexandre Freitas da Silva, Antonio Marinho da Silva Neto, Cleber Furtado Aksenen, Pedro Miguel Carneiro Jeronimo, Filipe Zimmer Dezordi, Suzana Porto Almeida, Hudson Marques Paula Costa, Richard Steiner Salvato, Tulio de Lima Campos, Gabriel da Luz Wallau, on behalf of the Fiocruz Genomic Network

**Affiliations:** Departamento de Entomologia, Instituto Aggeu Magalhães (IAM)-Fundação Oswaldo Cruz-FIOCRUZ, Recife, Pernambuco 50670-420, Brazil; Núcleo de Bioinformática (NBI), Instituto Aggeu Magalhães (IAM)-Fundação Oswaldo Cruz-FIOCRUZ, Recife, Pernambuco 50670-420, Brazil; Data Analysis and Engineering, Genomic Surveillance Unit, Wellcome Sanger Institute, Wellcome Genome Campus, Hinxton, Cambridgeshire CB10 1SA, UK; Fundação Oswaldo Cruz (Fiocruz) - Fiocruz-CE, Eusebio, Ceará 61760-000, Brazil; Fundação Oswaldo Cruz (Fiocruz) - Fiocruz-CE, Eusebio, Ceará 61760-000, Brazil; Departamento de Entomologia, Instituto Aggeu Magalhães (IAM)-Fundação Oswaldo Cruz-FIOCRUZ, Recife, Pernambuco 50670-420, Brazil; Núcleo de Bioinformática (NBI), Instituto Aggeu Magalhães (IAM)-Fundação Oswaldo Cruz-FIOCRUZ, Recife, Pernambuco 50670-420, Brazil; Fundação Oswaldo Cruz (Fiocruz) - Fiocruz-CE, Eusebio, Ceará 61760-000, Brazil; Núcleo de Bioinformática (NBI), Instituto Aggeu Magalhães (IAM)-Fundação Oswaldo Cruz-FIOCRUZ, Recife, Pernambuco 50670-420, Brazil; Secretaria Estadual da Saúde do Rio Grande do Sul, Centro Estadual de Vigilância em Saúde, Laboratório Central de Saúde Pública, Porto Alegre, Rio Grande do Sul 90450-190, Brazil; Núcleo de Bioinformática (NBI), Instituto Aggeu Magalhães (IAM)-Fundação Oswaldo Cruz-FIOCRUZ, Recife, Pernambuco 50670-420, Brazil; Departamento de Entomologia, Instituto Aggeu Magalhães (IAM)-Fundação Oswaldo Cruz-FIOCRUZ, Recife, Pernambuco 50670-420, Brazil; Núcleo de Bioinformática (NBI), Instituto Aggeu Magalhães (IAM)-Fundação Oswaldo Cruz-FIOCRUZ, Recife, Pernambuco 50670-420, Brazil; Department of Arbovirology, Bernhard Nocht Institute for Tropical Medicine, WHO Collaborating Center for Arbovirus and Hemorrhagic Fever Reference and Research, National Reference Center for Tropical Infectious Diseases, Bernhard-Nocht-Strasse 74, D-20359 Hamburg, Germany

## Abstract

ViralFlow v1.0 is a computational workflow developed for viral genomic surveillance. Several key changes turned ViralFlow into a general-purpose reference-based genome assembler for all viruses with an available reference genome. New virus-agnostic modules were implemented to further study nucleotide and amino acid mutations. ViralFlow v1.0 runs on a broad range of computational infrastructures, from laptop computers to high-performance computing (HPC) environments, and generates standard and well-formatted outputs suited for both public health reporting and scientific problem-solving. ViralFlow v1.0 is available at: https://viralflow.github.io/index-en.html.

## Introduction

Detecting and monitoring the spread of viral pathogens in the human population plays a crucial role for informing public health policies (1–3). Pathogen molecular surveillance is key to rapid detection and evaluation of emerging and reemerging lineages with altered phenotype. In the last years pathogen genome-wide surveillance has emerged as a powerful tool allowing higher resolution characterization of complete viral genomes and hence a more complete understanding of viral evolution and the emergence of new viral strains (4–6). The most recent example of the unprecedented amount of viral genomic data and their usefulness for public health comes from the COVID-19 pandemic where severe acute respiratory syndrome coronavirus 2 (SARS-CoV-2) has been monitored in near real-time across the globe (5,7). Genomic data were used to understand SARS-CoV-2 transmission dynamics and provide key information for the development of effective diagnostics and vaccines (8,9).

High-throughput sequencing (HTS) technologies have enabled dozens of pathogen genomes to be sequenced at once. This has moved the genomic surveillance bottleneck away from sequencing hundreds of genomes to analyzing ever-growing sequence datasets. Genomic surveillance of viral pathogens, like any surveillance system, benefits from rapid and consistent data sharing. However, managing, analyzing and summarizing the results of hundreds of genomes in a timely manner (daily/weekly basis) is a daunting task using separate software solutions. Bioinformatic challenges encompass several computational steps including multiple quality checks and intermediate steps that generate a substantial number of files requiring local processing and storage. To facilitate and accelerate the obtention of the genomic surveillance results, a number of pipelines/workflows have been proposed for processing large-scale SARS-CoV-2 genomic data such as HAVoC (10), ASPICov (11) and Viralrecon (https://doi.org/10.5281/zenodo.3901628). Additionally, there are a diverse range of tools available for general viral analysis such as V-pipe (12), ViReflow (13), Lazypipe (14), TRACESPipe (15), QVG (16), Haploflow (17), ASPIRE (18) and others. Some of these tools have been developed to handle viral metagenomic data or developed only for specific viruses, employing different assembly algorithms, each with its own advantages and limitations. ViralFlow v.0.0.6 is one of those that encapsulates several tools specifically tailored for genomic surveillance of SARS-CoV-2 (19). ViralFlow has been extensively used to assemble and analyze SARS-CoV-2 short-read genomic data and is the most used workflow in Brazil reported on GISAID, surpassing many commercial options (29.64% of genomes deposited with assembly methodology described on October 31, 2023). However, despite being widely used, some limitations still exist for broader genomic surveillance applicability including other viruses.

In this application note, we describe ViralFlow v1.0 and compare it with its predecessor. This new version was refactored and incorporated into a workflow language (NextFlow) that provides several advantages such as better management, an efficient parallelism and continuous checkpoint of processes. These features improve reproducibility and allow a rapid and easy implementation of new features to the workflow. Furthermore, this new version includes additional new agnostic software for mutation analysis and visualization that allows to more in depth characterize the effects of mutations on viral genomes. ViralFlow 1.0 also generates specific files containing viral-only sequenced reads, ensuring viral-only data sharing. In summary, this new version of ViralFlow is a general reference-based viral genome assembler allowing easy customization to any virus with a reference genome available.

## Materials and methods

ViralFlow v0.0.6 code was refactored within a NextFlow workflow system (20) focusing on four important pillars: (i) modularity; (ii) simplified installation steps and documentation; (iii) reproducibility; and (iv) code development and usability transparency. To achieve these aims, we used the ‘module’ functionality of NextFlow to separate the main tasks of ViralFlow v1.0 using Singularity containers (21). This modular aspect allows facilitated integration of new modules, and/or module replacements. Reduced numbers of command line steps are required to install ViralFlow v1.0 in both Ubuntu and MacOs systems (https://viralflow.github.io/index-en.html). The current version of ViralFlow v1.0 offers user flexibility to run analyses in two modes: ‘sars-cov-2′ and ‘custom’. The ‘sars-cov-2′ mode is pre-configured to execute all analyses without the need for configuring a reference genome. In this mode, ViralFlow v1.0 relies on the SARS-CoV-2 reference genome (NC_045512.2), which is automatically set up upon selection. The ‘custom’ mode allows for more flexibility, enabling the workflow to analyze any virus with an available genome. In this mode, users can specify the reference genome by either providing the NCBI accession number (–refGenomeCode parameter) for automatic download and configuration or providing a fasta file containing the reference genome (–referenceGenome, parameter), accompanied by an additional GFF annotation file (–referenceGFF parameter) which bears gene annotation needed for further analysis. Users can configure additional parameters according to their needs, e.g. activating (true) or deactivating (false) functions such as the prediction and annotation of mutations using SnpEff (–runSnpEff parameter) and outputting the mapped reads (–writeMappedReads parameter). Performance parameters can also be adjusted, including ‘–nextflowSimCalls’ to set the number of simultaneous calls that NextFlow will handle, as well as ‘–fastp_threads’, ‘–fastp_threads’ and ‘–mafft_threads’ parameters, which are set to use a single thread by default but can be adjusted for improved performance, if necessary. As ViralFlow v1.0 is based on NextFlow, users can invoke it directly using the main workflow module located at ‘∼/ViralFlow/vfnext/main.nf’ or utilize the ViralFlow wrapper configured within the ViralFlow environment along with the adjusted parameters file. Comprehensive documentation and running mode examples are available on the ViralFlow website.

The workflow processes raw reads based on a minimum phred score of 20 for quality control and employs a minimum length size (–minLen parameter) of 75 bases for reads trimming. This parameter may be adjusted by the user. The sequencing adapters are automatically detected and removed using fastp v0.23.4 (22) that allows deduplication of reads (–dedup parameter) to be performed, if desired. ViralFlow v1.0 removes primer regions more efficiently by employing the samtools ampliconclip from samtools v.1.11 (23) using a BED file containing primer positions provided by the user. Alternatively, a used refined number of bases can be removed from all reads using a ‘–trimLen’ parameter. Reads passing filters are mapped to a reference genome using BWA v.0.7.17 (24) in a default mode. Quality metrics for the alignment (BAM) file are calculated using Picard v.2.27.2 utilizing a default mapping and base quality of 30 that may be adjusted by the user (–base_quality and –mapping_quality parameters). Coverage plots are generated using the BAMdash tool (https://github.com/jonas-fuchs/BAMdash). The consensus genomes (fasta) are built using the most frequent allele per position, with samtools v.1.11 and iVar v1.4.2 (25), implementing a minimum depth to call consensus of 25 that may be adjusted by the user through the ‘–depth’ parameter. Bam-readcount v1.0.1 (26) and MAFFT v7.505 (27) are used to identify intrahost (minor allele) variants and build an alternative consensus (bearing the minor allele) using an in-house Python script. Single nucleotide polymorphism visualization plots are also generated using the snipit tool (https://github.com/aineniamh/snipit).

We implemented a new module containing freebayes v0.9.21 (28) and snpEff v.5.0 (29) to annotate and predict the impact of mutations detected. ViralFlow reports a plethora of files for each sample which includes annotated variants, mutation files, consensus genomes with major and minor alleles as well as with ambiguous characters, an alignment of the consensus and the reference, intrahost single nucleotide variants, mapped reads, genome assembly statistics, quality control reports, alignment files (BAM), plots showing mutations and genome coverage. Finally ViralFlow generates compiled output reports that include summarized information such as lineage, coverage breadth and depth that are generated in text/tabular format to allow further data wrangling.

To benchmark and evaluate the new ViralFlow v1.0 features, we compared its performance with that of ViralFlow v0.0.6 using four benchmark datasets with variable numbers of samples. Furthermore, we tested ViralFlow v1.0 performance using real datasets for SARS-CoV-2, monkeypox virus (MPXV), Dengue virus serotypes 1 and 2 (DENV-1 and DENV-2) and Zika virus (ZIKV).

### Illumina simulated reads

ART (30) was employed to generate simulated datasets using various high-quality SARS-CoV-2 genomes (horizontal coverage > 99%) from different lineages encompassing different sets of mutations (substitutions and indels) utilizing the ART-MountRainier-2016–06-05 software (https://github.com/scchess/Art/tree/master). We generated different numbers of paired FASTQ files: 1 FASTQ, 8 FASTQs, 16 FASTQs and 32 FASTQs. The artificial reads samples were created using the command **‘**art_illumina -ss HS25 -sam -i ’$file‘ -p -l 150 -f 500 -m 200 -s 0 -o ’$output". The -ss HS25 parameter stands for simulated HiSeq 2500 system, while the -p parameter stands for paired reads. Read length was set to 150 bp (-l 150 parameter) and -f 500 for 500 bp fragment size, with an -m 200 setting the standard deviation of the fragment size to 200 bp. The list of the genomes used for generating these artificial reads is accessible in Supplementary Table S1.

### Real-world benchmarking datasets

Benchmark datasets were obtained from https://github.com/CDCgov/datasets-sars-cov-2 and used to assess the performance of the workflows. These datasets consist of two real-life sets of reads and one negative control set, namely Bench 1 (BM data1), Bench 5 (BM data5) and Bench 6 (BM data6, negative control set), offering diverse perspectives on SARS-CoV-2 genomic data. BM data1, or the ‘Boston Outbreak’, encompasses 63 samples using Illumina metagenomic sequencing to understand real outbreak transmission. In BM data5, ‘Non-VOI/VOC Lineages’, 39 samples are used to benchmark non-specific lineage-calling workflows on Illumina, employing various primer sets, including Arctic v1, Arctic v3 and a random primer from NexteraXT. Lastly, BM data6, the ‘Failed QC’, features 24 samples serving as controls for bioinformatics quality control testing on Illumina, utilizing primer sets Arctic v3 and CDC in-house multiplex polymerase chain reaction (PCR) primers. The analyses of all datasets were run in triplicate, and the mean and standard deviation of the computational resources, maximum memory peak and runtime used were calculated.

### Computer setup

Two different computational platforms, namely AWS and IAM Carlos Chagas Cluster, were employed to simulate diverse execution setups of the workflow. The configuration of the Carlos Chagas Cluster node used comprised a Ubuntu Server 20.04.6 LTS containing 191 Giga bytes (GB) of RAM memory, dual Intel(R) Xeon(R) Gold 5220R CPUs (2.20 GHz), resulting in a total of 96 threads. To emulate a personal computer environment, an AWS instance was utilized with the following specifications: Ubuntu Server 22.04 LTS as the operating system, paired with a virtual machine instance of m5.large (8 GB RAM + 2 vCPUs).

### Workflow setup

Both versions of the workflow were executed with carefully matched parameters to ensure the fairest comparison possible considering the different software implemented. The specific arguments employed for each run can be found in Supplementary Table S2. While the two versions may differ in some steps, we extract a number of metrics such as: the maximum resident set size (i.e. the highest amount of physical memory the process utilized at any given moment) and wall-clock time.

Additionally, to validate the genome assembly of ViralFlow v1.0, we incorporated essential genomic statistics such as coverage breadth, coverage depth and the level of agreement between the assemblies (by comparing the lineages generated by each version of ViralFlow) and the corresponding consensus of the original samples.

### Diverse viral datasets

We performed both SARS-CoV-2 and custom mode analysis using real data (available on ENA project:PRJEB71472) using only ViralFlow v1.0. Five datasets were employed comprising 1 277 SARS-CoV-2, 56 MPXV, 37 DENV-1, 29 DENV-2 and 271 ZIKV samples. The performance metrics were collected from NextFlow using the parameter -with-trace.

## Results

### New features

This novel version of ViralFlow v1.0 provides new implementations and improvements compared with its previous version, v0.0.6. The main difference is the refactoring to the NextFlow workflow language, which has enabled enhancements in various aspects such as efficient parallelism and scalability of the workflow. Furthermore, with this new version one can analyze any virus with an available genome. Improvements have been made to handle amplicon sequencing data effectively by implementing primer removal based on a BED file, and deduplication reads have also been implemented using fastp. The validation tests conducted with and without deduplication demonstrate consistent results across versions and modes for coverage breadth and depth (Supplementary Table S3). Minor differences were observed in total reads and coverage depth when employing deduplication of reads, as expected (Supplementary Table S3). New analyses modules have been implemented for the annotation and prediction of mutation effects, as well as for their visualization and the generation of genomic maps with coverage breadth and depth. We introduced a package manager based on Micromamba for speed and robustness, along with standardizing containers to favor fast configuration and ensure the reproducibility of any analysis performed on ViralFlow v1.0.

### Viral genomic statistics

**Figure 1.**
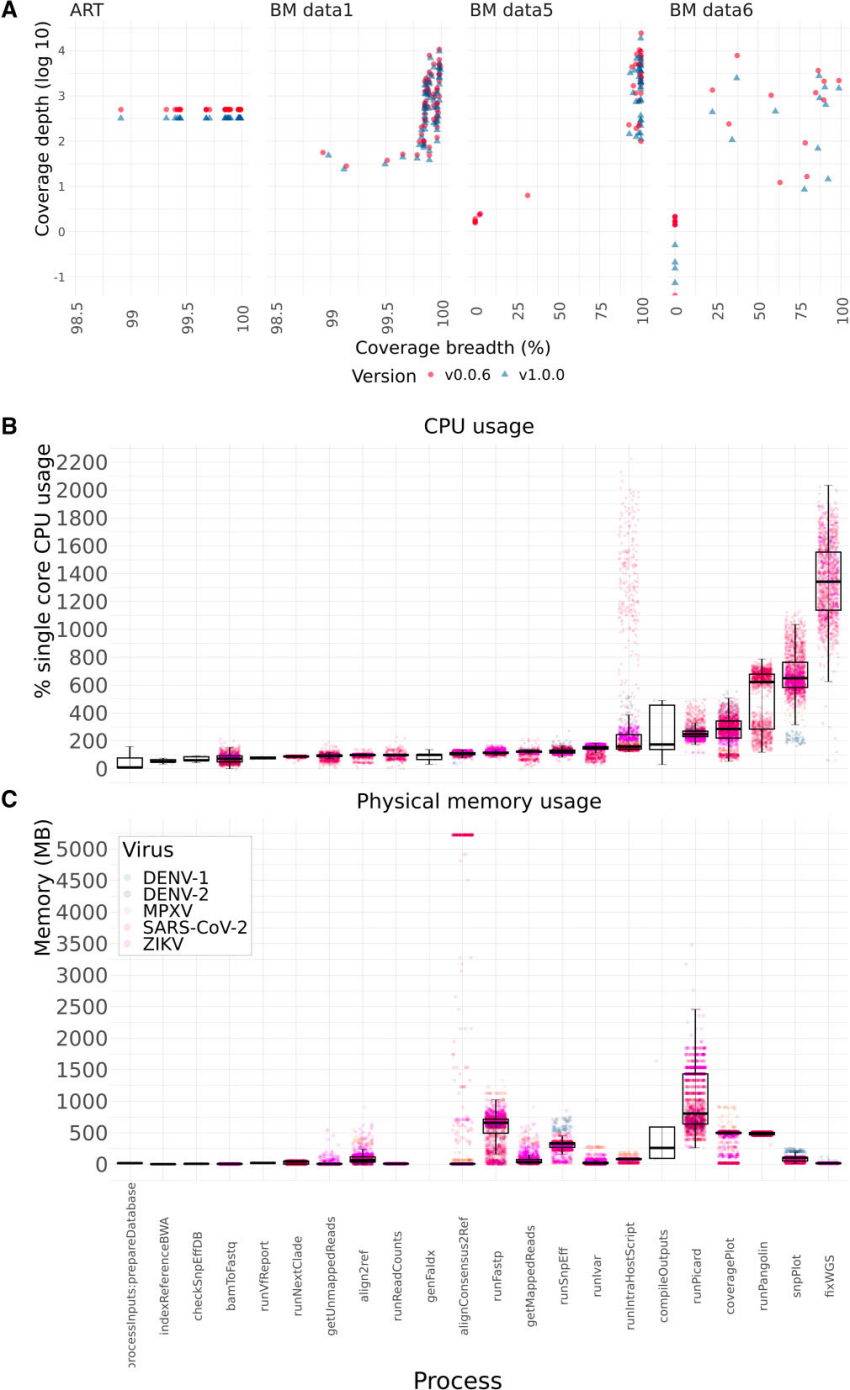
ViralFlow 0.0.6 and 1.0 performance evaluation. (**A**) Coverage depth and breadth of artificial and benchmark datasets. Statistic metrics recovered from viral assembly performed on the Carlos Chagas cluster. (**B**) CPU usage of ViralFlow v1.0 using real data for different viruses. (**C**) Physical memory usage of ViralFlow v1.0 using real data for different viruses.

The breadth and depth coverage were consistent across all datasets comparisons (Figure 1A), and the negative control set (Bench 6) exhibited reduced coverage breadth and depth, as expected (Figure 1A).

Time and memory consumption of both ViralFlow versions were similar in the AWS and Carlos Chagas Cluster environment, with ViralFlow v1.0 using slightly more memory, probably due to the new modules implemented (Supplementary Figure S1A, B).

Regarding the lineage concordance analysis, using the simulated reads (ART dataset), 100% concordance was observed for both versions of ViralFlow. In contrast, for the Bench datasets, version 1.0 consistently demonstrated higher concordance regarding the lineages identified previously in the Bench5 dataset. For the Bench 1 dataset, the same level of concordance was reached for both 0.0.6 and 1.0 versions (Supplementary Figure S2).

### Performance of ViralFlow v1.0 using diverse viral datasets running on high-performance computing (HPC)

In order to validate ViralFlow v1.0 with a wide range of viruses, we performed a complete run with Illumina sequencing data from SARS-CoV-2, MPXV, DENV-1, DENV-2 and ZIKV on the Carlos Chagas cluster.

Single CPU core usage was always between 200% and 500%, which is equivalent to 2–5 threads being used simultaneously. Three virus-agnostic processes (fixWGS, snpPlot, for all modes and runPangolin, for SARS-CoV-2 mode) requested more CPU usage (Figure 1B). Less than 1 GB was requested for most processes, while few tasks required more memory (up to 5.2 GB), such as alignConsensus2Ref and runPicard (Figure 1C). All tasks did not exceed more than 1 min to be completed separately (Supplementary Figure S3A; Supplementary Table S4). The processes requiring more time were runPicard, runIntrahostScript and runPangolin, for the SARS-CoV-2 mode. The majority of tasks executed by ViralFlow took no more than 2.9 min on average. However, tasks required 18 min to be completed (runPicard) for larger viral genomes such as MPXV (Supplementary Figure S3B; Supplementary Table S4). The analyses of 56 MPXV samples required a total of 54 min and 27 s to be completed (Supplementary File S1), while the analyses of 1 277 SARS-CoV-2 samples required a total of 8 h, 31 min and 22 s (Supplementary File S2).

Furthermore, we performed three additional analyses in the custom mode using three arbovirus samples: 37 DENV-1, 29 DENV-2 and 271 ZIKV samples. ViralFlow v1.0 took 24 min (Supplementary File S3), 19 min and 28 s (Supplementary File S4) and 2 h 5 min and 8 s to run for each dataset, respectively.

## Discussion

Viral genomic surveillance has been incorporated into routine monitoring programs around the globe. However, the ease of generating raw viral sequences has not been readily accompanied by bioinformatic tools tailored for timely output analyses of hundreds to thousands of samples. Here we describe ViralFlow v1.0, a workflow that requires low memory and CPU consumption, and hence can be implemented in low resource settings. This workflow can also be scalable in HPC environments. Moreover, ViralFlow v1.0 has improved modularity, transparency and usability, as well as improved best practices for advanced users and developers. These improvements also allow increased reproducibility and performance. Despite the availability of several computational pipelines for viral genomic data analysis in the literature, few of them are implemented into workflow managers languages, and those that do offer such features are limited to specific viruses. ViralFlow, while sharing similarities with some pipelines, combines both workflow management and flexibility for any virus. We provided a feature comparison table, including ViralFlow and several other pipelines developed with similar goals in mind, that will be employed in future benchmark comparison using different case studies, genome assembly metrics and performance (Supplementary Table S5). Planned future developments will focus on incorporating heuristics for automatically defining best reference genome selection, which is particularly useful for non-segmented genomes such as influenza virus, and analysis of long reads.

## Supplementary Material

lqae056_Supplemental_Files

## Data Availability

The code is publicly available and maintained within a GitHub repository (https://github.com/WallauBioinfo/ViralFlow, also on Figshare, https://doi.org/10.6084/m9.figshare.24902754.v4), allowing version control, scrutiny and free use/reuse (MIT license). Data used in this manuscript were simulated or generated from original samples. All the data are available at ENA project: PRJEB71472.
