# Supplementary material for: ViralFlow v1.0—a computational workflow for streamlining viral genomic surveillance
Source: NAR Genom Bioinform. 2024 May 25;6(2):lqae056. doi: 10.1093/nargab/lqae056 (PMC11127631; doi:10.1093/nargab/lqae056)
Supplement: lqae056_Supplemental_Files [file lqae056_supplemental_files.zip › Supplementary figures.pdf]

ViralFlow v1.0.0 - a computational workflow for streamlining viral genomic surveillance

Alexandre Freitas da Silva*, Antonio Marinho da Silva Neto*, Cleber Furtado Aksenen*, Pedro Miguel Carneiro Jerônimo*, Filipe Zimmer Dezordi*, Hudson Marques Paula Costa, Richard Steiner Salvato, Tulio de Lima Campos#, Gabriel da Luz Wallau#

*These authors contributed equally to this manuscript and share the first authorship.
# These authors share the senior authorship.

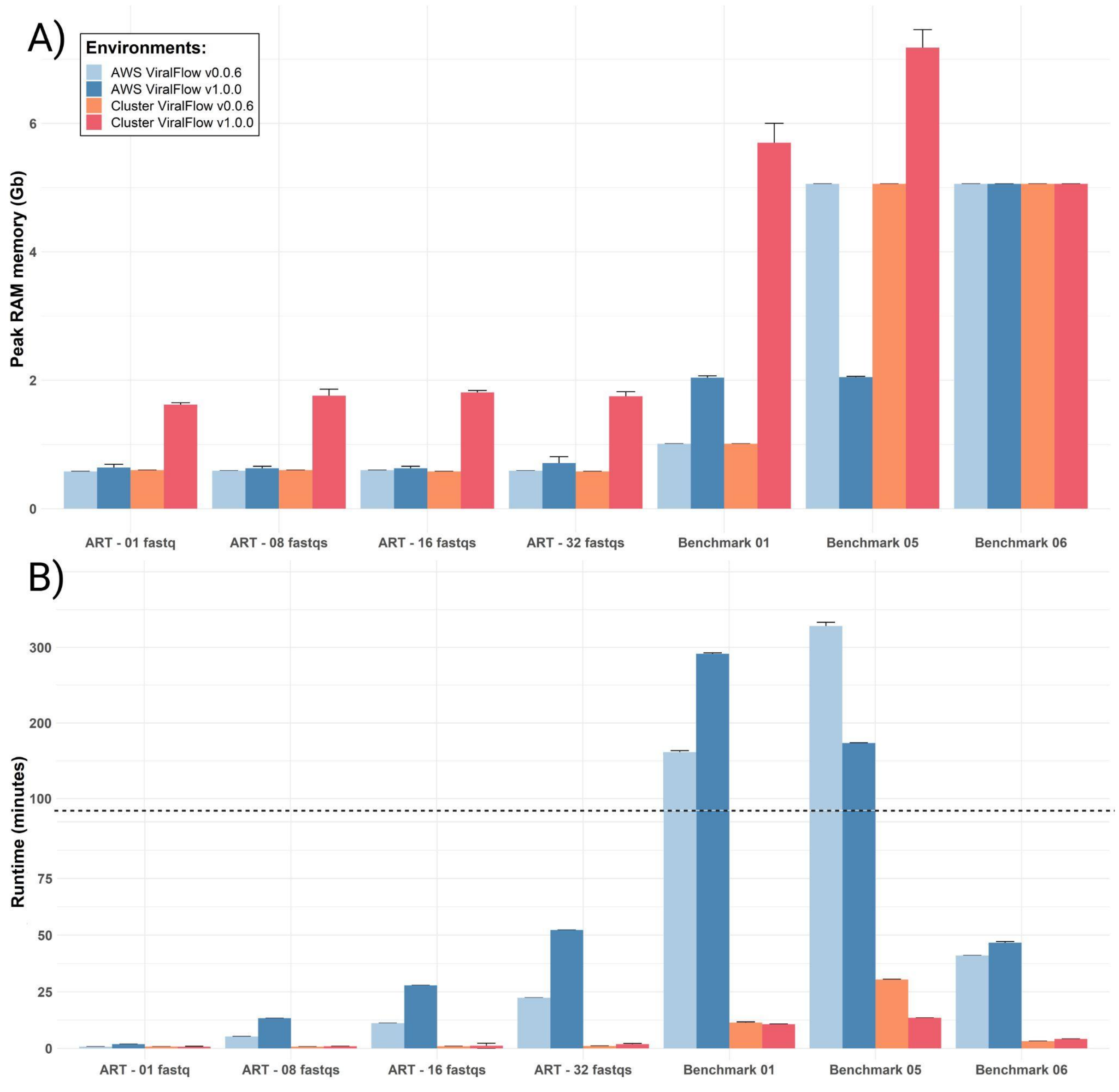

Supplementary Figure 1 - Maximum memory and runtime across iterations 0.0.6 and 1.0.0 of the ViralFlow workflow conducted through the AWS cloud infrastructure and Carlos Chagas HPC server. This figure provides a detailed comparison of peak RAM memory in gigabytes (A) and runtime in minutes (B), tested with different amounts of simulated illumina reads: 01 FASTQ; 08 FASTQs; 16 FASTQs and 32 FASTQs and benchmark datasets: 01;05 and 06.

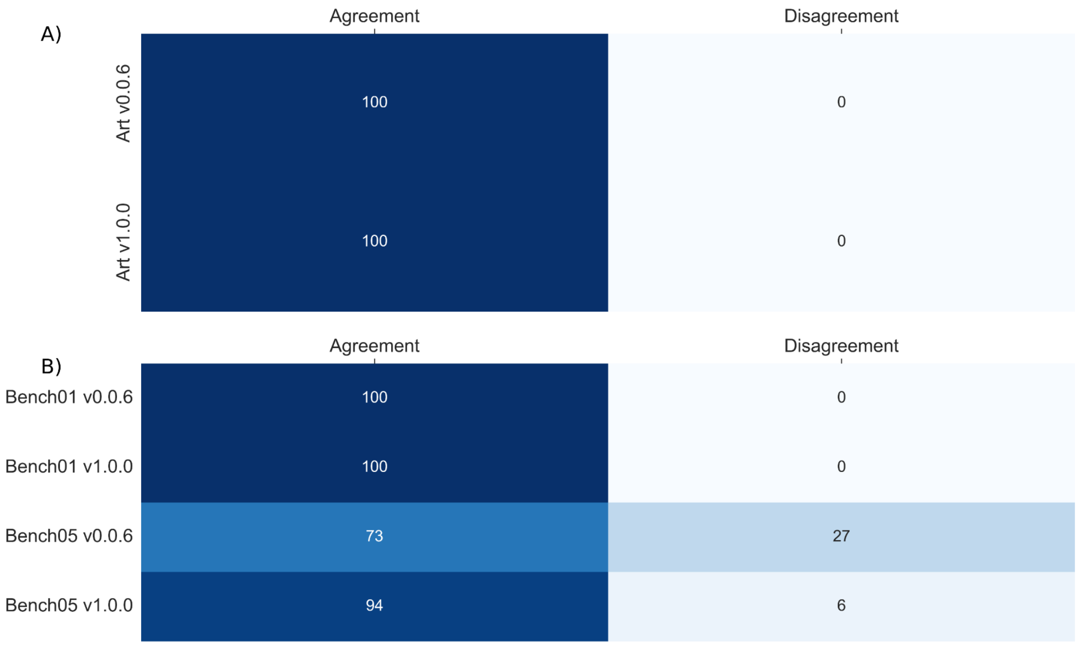

Supplementary Figure 2 - Percentage of agreement between ViralFlow pipeline versions 0.0.6 and 1.0.0 for various datasets: A) artificial reads generated from high coverage breadth sequences (> 99%), with comparison focused on the ability of ViralFlow to match the same lineage as the original high-quality sequences deposited at GISAID. B) Real dataset derived from confirmed outbreaks of SARS-CoV-2 cases, where the comparison involved the lineage assignment of the original consensuses used for benchmark and those assembled by each version of ViralFlow.

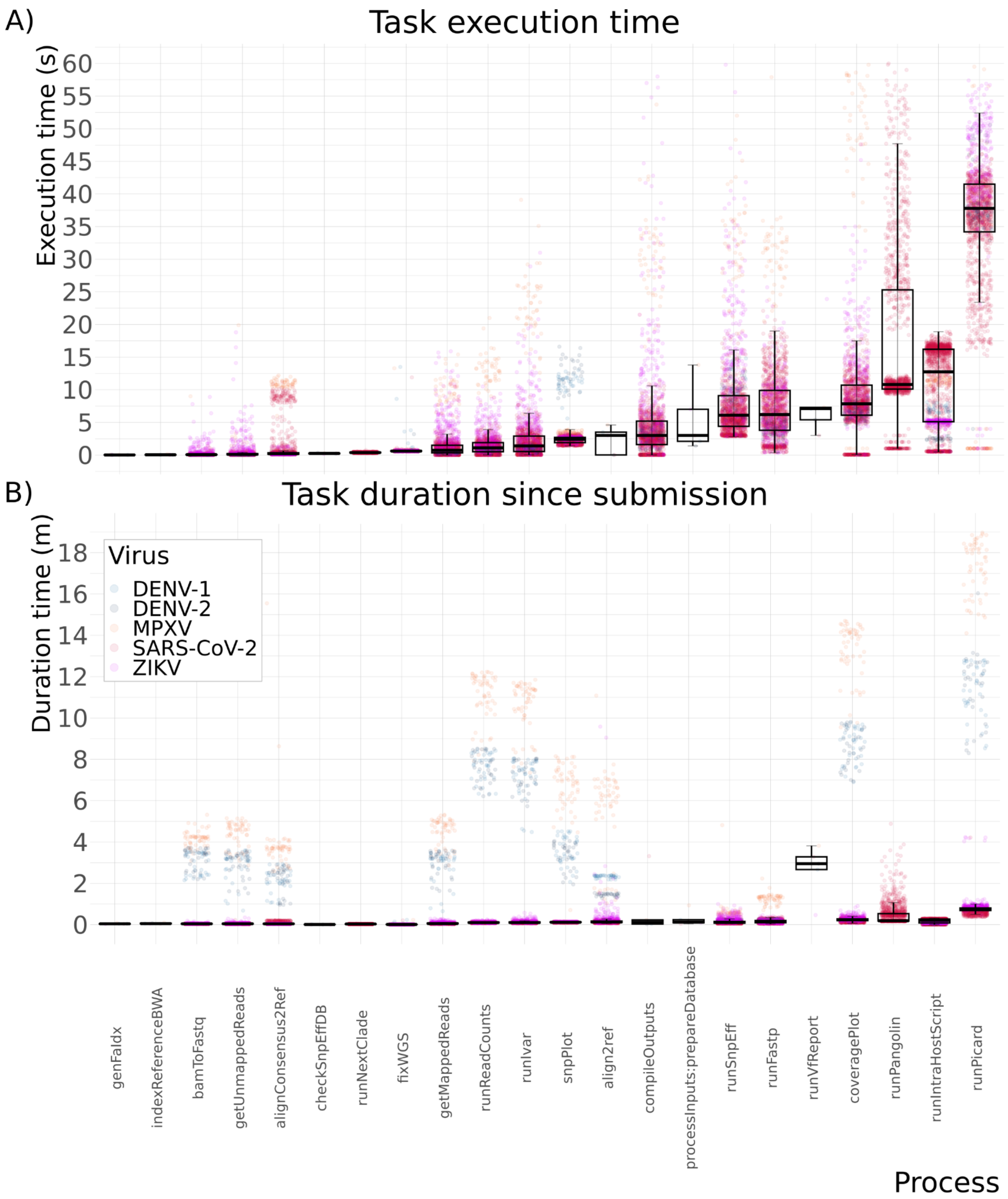

Supplementary Figure 3. Time per tasks performed on ViralFlow v1.0.0 for different viruses based on Nextflow trace file. A)Task execution time showing the realtime of the process. B)Task duration representing the time elapsed to complete the process since the submission. Colored points represent task execution time for each sample analyzed.
