# Supplementary material for: ViralFlow v1.0—a computational workflow for streamlining viral genomic surveillance
Source: NAR Genom Bioinform. 2024 May 25;6(2):lqae056. doi: 10.1093/nargab/lqae056 (PMC11127631; doi:10.1093/nargab/lqae056)
Supplement: lqae056_Supplemental_Files [file lqae056_supplemental_files.zip › Supplementary table 1.pdf]

SUPPLEMENTAL TABLE

Data Availability

GISAID Identifier: EPI_SET_231215sg
doi: 10.55876/gis8.231215sg

All genome sequences and associated metadata in this dataset are published in GISAID's EpiCoV database. To view the contributors of each individual sequence with details such as accession number, Virus name, Collection date, Originating Lab and Submitting Lab and the list of Authors, visit 10.55876/gis8.231215sg

Data Snapshot

- EPI_SET_231215sg is composed of 32 individual genome sequences.
- The collection dates range from 2021-01-02 to 2023-02-06;
- Data were collected in 8 countries and territories;
- All sequences in this dataset are compared relative to hCoV-19/Wuhan/WIV04/2019 (WIV04), the official reference sequence employed by GISAID (EPI_ISL_402124). Learn more at https://gisaid.org/WIV04.
