# Supplementary material for: ViralFlow v1.0—a computational workflow for streamlining viral genomic surveillance
Source: NAR Genom Bioinform. 2024 May 25;6(2):lqae056. doi: 10.1093/nargab/lqae056 (PMC11127631; doi:10.1093/nargab/lqae056)
Supplement: lqae056_Supplemental_Files [file lqae056_supplemental_files.zip › gisaid_info and Supplementary file 1-4.zip!/Supplementary file 1.html]

[peaceful\_kirch] Nextflow Workflow Report


Nextflow Report


- Summary
- Resources
- Tasks

[peaceful\_kirch]

# Nextflow workflow report

## `[peaceful_kirch]` *(resumed run)*

Workflow execution completed successfully!

Run times
:   05-Dec-2023 09:36:40 - 05-Dec-2023 10:31:06
    (duration: **54m 27s**)

790 succeeded

0 cached

0 ignored

0 failed

Nextflow command
:   ```
    nextflow run /home/alexandre/ViralFlow/vfnext/main.nf --virus custom --inDir /home/alexandre/paper_viralflow/mpox/ --outDir /home/alexandre/paper_viralflow/mpox/output_final/ --depth 25 --trimLen 30 --refGenomeCode MT903345.1 --writeMappedReads true --runSnpEff true -resume -profile fiocruz_default --nextflowSimCalls��96 -with-report report.html -with-trace -with-timeline
    ```

CPU-Hours
:   `4.9`

Launch directory
:   `/home/alexandre/paper_viralflow/mpox`

Work directory
:   `/home/alexandre/paper_viralflow/mpox/work`

Project directory
:   `/home/alexandre/ViralFlow/vfnext`

Script name
:   `main.nf`

Script ID
:   `e97c5d2e44022d90eccc0f23ce772fd4`

Workflow session
:   `183afbfb-87b2-47c9-ac6c-f3166d2a038c`

Workflow profile
:   fiocruz\_default

Nextflow version
:   version 21.10.6, build 5660 (21-12-2021 16:55 UTC)

## Resource Usage

These plots give an overview of the distribution of resource usage for each process.

#### CPU

- Raw Usage
- % Allocated

#### Memory

- Physical (RAM)
- Virtual (RAM + Disk swap)
- % RAM Allocated

#### Job Duration

- Raw Usage
- % Allocated

#### I/O

- Read
- Write

## Tasks

This table shows information about each task in the workflow. Use the search box on the right
to filter rows for specific values. Clicking headers will sort the table by that value and
scrolling side to side will reveal more columns.

Values shown as:

Human readable
Raw values

(tasks table omitted because the dataset is too big)

Generated by Nextflow, version 21.10.6
